# Supplementary material for: Are carbon emissions trading and green financial instruments synergistic? -Comprehensive quantitative research based on content analysis
Source: PLoS One. 2024 Mar 7;19(3):e0298601. doi: 10.1371/journal.pone.0298601 (PMC10919601; doi:10.1371/journal.pone.0298601)
Supplement: S1 Data — (ZIP) [file pone.0298601.s001.zip › Data/地级市Z_F工作报告.zip!/╜¡╦╒/╦╒╓▌╩╨/└┤╫╘╣1⁄2╓┌║┼í╛┬φ┐╦╩2╛▌═°í┐_2017.docx]

各位代表：

　　现在，我代表苏州市人民政府，向大会报告工作，请予审议，并请市政协各位委员和其他列席人员提出意见。

　　一、本届政府的工作回顾

　　本届政府在中共苏州市委的领导下，深入贯彻党的十八大、十八届三中四中五中六中全会和习近平总书记系列重要讲话特别是视察江苏重要讲话精神，紧紧围绕“五位一体”总体布局和“四个全面”战略布局，主动适应经济发展新常态，自觉践行新发展理念，团结依靠全市人民，克难奋进，开拓创新，推动经济社会发展取得了一系列重大成就。

　　五年来，经济保持平稳增长，产业结构不断优化，质量效益持续提高。地区生产总值、一般公共预算收入年均分别增长8.6%和9.5%。按常住人口计算人均地区生产总值由1.58万美元提高到2.2万美元。产业结构实现“三二一”战略性转变，新兴产业、高新技术产业产值占规模以上工业总产值比重分别提高11.7个和9.6个百分点，形成新材料、高端装备制造、软件和集成电路等千亿级新兴主导产业。科技综合实力保持全省第一，发明专利申请量、授权量位居全国城市前列。新型平板显示、纳米技术应用、生物医药等领域取得重大创新成果，昆山维信诺OLED项目荣获国家技术发明一等奖，亨通集团摘得中国工业大奖。

　　五年来，体制改革全面推进，开放创新不断深化，发展活力持续增强。新一轮政府机构改革顺利完成，商事制度改革举措加快落实，市场主体达到112.8万户，增加49.9万户。新增上市公司43家，累计达到113家。农村集体产权制度、医药卫生体制等改革取得重要进展。新增4个国家级开发区和4个综合保税区，苏州工业园区开放创新综合试验、昆山深化两岸产业合作试验区和国家外债宏观审慎管理试点成效显著。一般贸易出口占出口总额的比重提高到32.4%，实际使用外资390亿美元，中方境外实际投资额每年均居全省首位。第二届中非民间论坛、第四届中欧政党高层论坛、第四次中国—中东欧国家领导人会晤在我市成功举办。

　　五年来，城乡建设协同并进，一体进程不断加快，环境面貌持续改善。实施行政区划重大调整，城镇格局得到优化，常住人口城镇化率达到75.5%。城乡发展一体化综合改革深入推进，政策制度框架基本建立。城市轨道交通1号线2号线、中环快速路和七浦塘拓浚整治、城区“自流活水”工程等重大基础设施建成投用。主要污染物排放大幅度降低，PM2.5年均浓度比2013年下降34.3%，水环境状况持续好转。在全国率先实施生态补偿，“四个百万亩”得到严格保护。我市成为首个国家生态城市群，苏州市、昆山市成为首批国家生态园林城市，同里、梅李、甪直、震泽入选全国宜居示范小镇。

　　五年来，社会事业繁荣进步，公共服务不断完善，民生福祉持续增进。累计完成实事项目142项，总投资670亿元。帮助困难人员就业11.6万人，城镇登记失业率每年控制在2%左右，我市成为全国创业先进城市。城镇、农村居民人均可支配收入年均分别增长9.4%和10.2%。在全国率先实现城乡养老、医疗和居民最低生活保障“三大并轨”，企业退休人员人均基本养老金年均增长9.5%，最低生活保障标准由每人每月500元提高到810元。城乡社区卫生服务普及率、人口覆盖率保持100%。人均预期寿命由81.56岁提高到82.87岁。我市获批国家首批义务教育发展基本均衡市和公共文化服务体系示范区，入选“全球创意城市网络—手工艺与民间艺术之都”。成功举办第53届世界乒乓球锦标赛。苏州市、张家港市蝉联全国文明城市。我市实现全国社会治安综合治理优秀市“六连冠”，太仓市成为全省首获长安杯的县级市。

　　五年来，政府职能加快转变，自身建设不断加强，服务效能持续提升。大力削减行政审批事项，非行政许可审批事项全部取消。政府行政权力、政府部门责任等清单向社会公布，覆盖到村和社区的政务服务体系基本形成。累计取消和减免行政事业性收费186项。提请市人大常委会审议通过地方性法规9件，颁布政府规章14件、规范性文件41件。认真开展党的群众路线教育实践活动、“三严三实”专题教育、“两学一做”学习教育，转政风、严政纪，惩腐败、建机制，政府系统党风廉政建设全面加强。

　　各位代表，在刚刚过去的一年里，我们坚决贯彻中央、省和中共苏州市委的决策部署，认真落实市十五届人大五次会议确定的目标任务，大力推进供给侧结构性改革，切实抓好稳定增长、转型创新、改革开放、城乡建设、生态优化、民生改善等工作，经济实现平稳增长，转型升级步伐加快，社会保持和谐稳定，人民生活水平继续提高，“十三五”发展实现良好开局。

　　经济运行稳中提质。全市完成地区生产总值1.54万亿元（预计数，下同），增长7.5%以上；一般公共预算收入1730亿元，增长10.8%；社会消费品零售总额4937亿元，增长10.7%；全社会固定资产投资5648.5亿元，投资效益得到提升。工业经济平稳运行，实现规模以上工业总产值3.07万亿元。“三去一降一补”年度任务全面完成，关停淘汰落后低效产能企业977家，去库存与稳房价得到有效协调，新增企业直接融资1399亿元，降低企业成本超过300亿元，111个补短板项目完成投资200亿元以上。落实中国制造2025苏州实施纲要和打造先进制造业基地行动计划，友达光电6代线面板一期、三星液晶显示二期、力神动力锂电池一期等重大产业项目顺利竣工，工业机器人、光伏产业产值分别增长14.8%和10.3%，新兴产业产值占规模以上工业总产值比重达到49.8%。服务业量质齐升，增加值占地区生产总值比重达到51.4%，提高1.5个百分点。实现旅游总收入2078亿元，增长11.5%。我市获评中国旅游休闲示范城市。文化创意产业营业收入超过4700亿元，增长15%。阳澄湖数字文化创意产业园获批全国版权示范基地。完成金融业增加值1330亿元，增长13%。新业态新模式蓬勃发展，出台大数据产业发展规划和政策意见，阿里云计算苏州公司投入运营，华为苏州研究所开工建设，中兴物联网、京东物流中心和云小镇等落户苏州，电子商务交易额增长30%，软件和信息技术服务收入增长18.7%。壮大农业经营主体，培育新型职业农民，现代农业加快发展。新增高标准农田3.5千公顷、现代农业园区面积4.9千公顷，农业适度规模经营比重达到92%。

　　科技创新力度加大。实施打造产业科技创新高地行动计划和推进创新驱动发展“3+1”系列政策意见，着力建设苏南国家自主创新示范区核心区。全社会研究与试验发展经费支出占地区生产总值比重达到2.7%。新增国家高新技术企业920家，累计达到4133家，高新技术产业产值占规模以上工业总产值比重达到46.9%。加快构建创新载体，中国科学院电子所苏州研究院、纳米真空互联实验站开工建设，清华—苏州环境创新研究院、华中科技大学（苏州）脑空间信息技术研究院、牛津大学—苏州先进研究中心、悉尼大学中国中心相继设立，新增国家级企业技术中心2家，累计达到24家。加大人才培养引进力度，新增高层次人才超过2万人、高技能人才3.24万人，累计分别达到19.8万人和52.43万人；其中新增国家“千人计划”人才32人，累计达到219人。实施金融支持企业自主创新行动计划，全市创投管理资金规模达到1050亿元，通过苏州综合金融服务平台实现企业融资1550亿元，其中无抵押贷款超过300亿元。加强知识产权保护运用，万人有效发明专利拥有量达到37.6件，国际PCT专利申请量950件；新增驰名商标5件，累计达到117件；江苏国际知识产权运营交易中心获批设立，苏州高新区成为国家医疗器械专利导航产业发展实验区和首批知识产权服务业集聚发展示范区。我市被评为全国质量强市示范城市。

　　改革开放深入推进。以释放市场活力为主线，深化简政放权改革。取消、下放行政审批事项44项，相对集中行政许可权改革稳步推进，苏州工业园区试点经验在全省推广。商事制度改革不断深化，新增各类市场主体20万家、注册资本5170亿元，分别增长19.5%和43.6%。营业税改征增值税试点任务如期完成，不动产统一登记全面实施。苏州民营资本投资控股有限公司成立运营。市属国有企业分类监管考核有效实施。苏州资产管理公司获批建立，苏州股权交易中心、江苏（苏州）环境资源交易中心建成运行。

　　以促进投资和贸易便利化为主线，深化开放型经济体制改革。我市成为国家跨境电子商务综合试验区、服务贸易创新发展试点城市，常熟服装城获批开展市场采购贸易，昆山综合保税区、苏州工业园综合保税区获得企业增值税一般纳税人资格试点，内外贸一体化改革取得突破。51项自由贸易试验区改革试点经验在我市复制推广，跨境人民币业务试点政策在全市共享。完成进出口总额1.81万亿元，一般贸易比重提高4.2个百分点，服务贸易增长15%，贸易高端化步伐加快。对“一带一路”沿线国家和地区的出口比重达到20.3%，我市被列为“中欧班列”中枢节点城市。实际使用外资完成60亿美元的目标任务，新增具有地区总部特征或共享功能的外资企业30家。中方境外协议投资额达到32亿美元，增长56.7%。中国—中东欧国家最高法院院长会议和卫生部长论坛在我市成功举办，新增国际友城3个。对台、侨务工作再创新业绩。对口支援、南北共建、扶贫协作取得新成效。

　　城乡发展更趋协调。土地利用总体规划调整完善，重点村特色村规划以及一批历史文化街区规划、试点地块城市设计编制完成。常嘉高速公路、张家港疏港高速公路建成通车，沪通铁路苏州段建设进展顺利。苏申内港线、外港线航道整治积极开展。苏州港货物吞吐量达到5.8亿吨，增长7.3%。城市轨道交通加快建设，2号线延伸线投入试运营，新增运营里程15.6公里。南环快速路西延、228省道北段竣工通车，市区人民路综合整治提升工程基本完成。苏州汽车西站综合客运枢纽建成投运，成为华东地区最大的运贸综合体。建成换乘停车场24处、泊位1万多个。苏州工业园区桑田岛地下综合管廊建成使用，海绵城市试点项目积极推进。我市实现全国人民防空先进城市“四连冠”。1000千伏特高压淮上线东吴变电站投入运行。“城市光网”覆盖到村，“智慧苏州”加快建设。扎实开展“城市环境大家管、市容秩序大提升”等综合治理行动，城市面貌得到改善。

　　农村改革纵深推进，股权固化改革、土地承包经营权确权登记颁证工作基本完成。城乡发展一体化基金规模和引导效应持续放大，支农惠农成效明显。农村集体总资产达到1720亿元，增长6.8%；村均稳定性收入超过800万元，增长3.2%；所有村年稳定性收入超过200万元的目标顺利实现。完成1580个自然村庄生活污水治理任务，农村生活污水处理率达到75%，提高15个百分点。美丽镇村建设步伐加快，首批16个示范镇建设任务全面完成，新增美丽村庄示范点10个、三星级康居乡村305个，甪直、震泽入选首批中国特色小镇。

　　生态环境有效改善。优化生态保护红线区域，新增保护面积54.5平方公里。生态文明建设“十大工程”加快实施，完成投资127.1亿元。狠抓大气污染治理，推进重污染企业提标改造和重点行业挥发性有机物深度治理，加快淘汰燃煤锅炉、黄标车，加强扬尘污染防控，PM2.5年均浓度下降20.7%，空气质量优良天数比例提高2.1个百分点。太湖流域水环境综合治理深入实施，阳澄湖新三年生态优化行动开局良好，集中式饮用水源地水质达标率保持100%。土壤污染调查和修复工作积极开展，噪声污染防治、核与辐射安全管控、危险废弃物和建筑垃圾规范处置不断加强。强化农业面源污染治理，农业废弃物无害化处理和资源化利用率达到97.6%。启动生活垃圾处置系列工程，新增生活垃圾分类和资源化综合利用试点小区、村154个。市区新增绿地350万平方米。成功举办第九届江苏省园艺博览会和第十届国际湿地大会。坚持源头控污，推进节能减排，单位地区生产总值能耗下降、主要污染物排放总量削减完成省下达任务。国际能源变革论坛永久会址落户我市，常熟经济技术开发区入选国家级循环化改造试点示范园区。

　　社会建设全面加强。增加教育资源供给，新建和改扩建中小学、幼儿园52所，新增学位6.6万个。完善基础教育评价体系，提高师资队伍水平，出台乡村教师支持计划，实施民办学校校安工程，促进义务教育优质均衡发展。健全现代职业教育体系，校企合作、产教融合更加紧密。推进名城名校融合发展，一批合作项目启动实施。医药卫生体制改革取得成效，分级诊疗制度初步形成，上下联动、统一协作的健康管理综合服务机制逐步健全。建成紧密合作型医联体97个，组建家庭医生团队1030个，签约家庭65万户，实行社区药品“直通车”制度。苏州药品口岸检验所获批建立。建成国家级重大疾病救治中心10家，苏州科技城医院投入使用。太仓市获得世界卫生组织健康城市最佳实践奖。完善计划生育管理服务，“全面两孩”政策平稳实施。加快文化设施建设，苏州现代传媒广场、苏州高新区文体中心、吴中区公共文化中心顺利建成。文化遗产保护得到加强，江南水乡古镇和海上丝绸之路申遗工作积极开展，苏州非物质文化遗产展示馆建成使用。完成全市地方志二轮编纂工作，苏州丝绸档案入选世界记忆亚太地区名录。苏州工业园区体育中心抓紧建设，苏州湾体育公园建成开园，环古城河健身步道全面提升。苏州体育健儿在里约奥运会、残奥会上夺得7枚金牌。成功举办“汤尤杯”世界羽毛球团体锦标赛和市第十四届体育运动会。民族团结进步事业不断发展，宗教工作进一步加强。全市第六届社区居委会和第十一届村委会换届选举圆满完成。落实各项优抚政策，支持驻苏部队建设，我市实现全国双拥模范城“六连冠”。

　　深化文明城市长效建设，社会文明程度得到提升。基层协商民主建设稳步推进，社区服务社会化、全科社工等试点有序实施。太仓市“政社互动”实践项目名列全国社区治理十大创新成果之首。社会信用体系数据库和服务平台顺利建成，我市信用指数位居全国地级市首位。出台户籍准入管理办法，强化流动人口积分管理与服务。严格落实安全生产责任制，深入开展突出问题专项整治，加大隐患排查整改和监管执法力度，坚决防范和遏制重特大事故，安全生产形势总体平稳。依法分类处理群众信访诉求，一批信访矛盾得到化解。健全立体化信息化社会治安防控体系，圆满完成G20峰会等重大活动期间的安保任务，社会保持平安稳定。应急管理体系更加完善，应急保障能力持续增强。公共法律服务水平不断提升，各级政府和村、社区法律顾问制度全面建立，我市获评全国“六五”普法先进城市。

　　民生质量稳步提高。城镇、农村居民人均可支配收入分别达到5.44万元和2.78万元，增长8%和8.5%。年度实事项目全面完成。新增就业17.11万人，苏州籍应届高校毕业生就业率达到98.5%。苏州工业园区成为全国构建和谐劳动关系综合试验区。社会保障水平继续提高，市区企业退休人员基本养老金人均每月增加148元，城乡居民基础养老金提高到每人每月430元。社会救助体系逐步健全，临时救助范围从户籍人口扩大到持居住证的常住人口。苏州公益慈善综合指数位居全国城市前列，4个县级市全部入围县域十强。新增养老机构床位3066张，日间照料中心89家，虚拟养老院服务覆盖到社区和村。新开工建设保障性住房3.14万套。新增缴存住房公积金职工68.85万人。国家“公交都市”创建工作深入开展，市区新购公交车670辆，新辟公交线路34条、社区巴士线路20条。落实粮安工程建设三年行动计划，粮食安全保障能力进一步增强。市区居民消费价格总水平涨幅2.7%。食品药品安全放心行动扎实开展，建成覆盖城乡的食品安全监测预警体系。

　　各位代表，过去的五年，发展历程极不平凡，发展成就令人鼓舞。过去的一年，实现“十三五”发展良好开局，实属不易。这是全市人民在中共苏州市委正确领导下凝心聚力、团结奋斗的结果，是市人大、市政协加强监督、鼎力支持的结果。在此，我代表市政府向全市人民，向人大代表、政协委员，向离退休老同志，向各民主党派、工商联和无党派人士，向各人民团体，向驻苏解放军、武警和消防官兵，向国家和省驻苏单位，向参与、支持、关心苏州各项事业发展的海内外朋友，表示衷心的感谢和崇高的敬意！

　　在看到成绩的同时，我们也清醒地认识到经济社会发展和政府工作仍然存在不少问题，主要是：经济下行压力依然较大，工业企业投入不足，部分企业生产经营困难；产业层次整体不够高，科技创新还未成为推动发展的主动力；供给侧结构性改革还需深化；资源环境约束趋紧，生态保护、污染防治、节能减排工作仍需持续用力；民生保障、社会治理、基础设施等领域存在不少短板，统筹城乡发展还要加大力度；作风效能建设有待加强，政府工作人员特别是领导干部的能力水平需要进一步提高。对于这些问题，必须采取更为有效措施，努力加以解决。

　　二、新一届政府工作的总体思路和奋斗目标

　　根据中共江苏省第十三次党代会和苏州市第十二次党代会精神，建议新一届政府工作的总体思路是：高举中国特色社会主义伟大旗帜，以邓小平理论、“三个代表”重要思想、科学发展观为指导，全面贯彻党的十八大、十八届三中四中五中六中全会精神和习近平总书记系列重要讲话特别是视察江苏重要讲话精神，紧紧围绕“五位一体”总体布局和“四个全面”战略布局，自觉践行新发展理念，适应把握引领经济发展新常态，牢牢把握稳中求进工作总基调，扎实推进供给侧结构性改革，紧扣聚力创新、聚焦富民、高水平全面建成小康社会，大力实施创新驱动、民生优先、生态改善、城乡一体、开放提升、文化繁荣战略，争当建设经济强、百姓富、环境美、社会文明程度高新江苏的先行军排头兵。

　　高水平全面建成小康社会的核心要义是发展水平要更高、群众获得感要更强。围绕这一要求，我们要全力建成走在全省前列、体现苏州水平、惠及广大人民群众的小康社会。要聚力创新，牢牢把握创新发展、转型发展的时间窗口，坚持问题导向、目标导向，把主要精力、主要资源、主要政策用在自主创新特别是原始性创新上，形成具有苏州标志的创新高峰。要聚焦富民，认真贯彻习近平总书记关于民生工作“七个更”的重要指示，把保障和改善民生作为一切工作的出发点和落脚点，顺应民生需求新变化，落实民生工作新举措，提高广大群众的富裕程度和生活质量。以“两聚一高”为发展取向、工作导向和奋斗指向，按照建成具有国际竞争力的先进制造业基地、具有全球影响力的产业科技创新高地、具有独特魅力的国际文化旅游胜地和具有较强综合实力的现代化国际化大城市的发展定位，建议新一届政府的主要奋斗目标是：

　　——经济发展质量显著提升。经济保持中高速增长，产业迈向中高端水平。“一基地一高地”建设取得明显成效，科技创新成为经济发展主动力，现代产业体系基本建立。开放型经济提质增效，对外开放的深度和广度进一步拓展。提前实现地区生产总值和城乡居民人均收入比2010年翻一番，居民收入、企业利润、财政收入“三个口袋”更加充实。

　　——人民生活水平显著提升。全民共享、共建共享加快推进，实现基本公共服务均等化便捷化优质化，基本形成与步入高收入阶段和成熟社会阶段相适应的公平分配格局，人民群众普遍享有更好的教育、更稳定的工作、更满意的收入、更可靠的社会保障、更高水平的医疗卫生服务、更舒适的居住条件、更优美的环境。

　　——生态建设成效显著提升。坚决打好大气、水、土壤污染防治三大战役，生态保护治理取得重大成果，突出环境问题得到切实解决。能源资源利用效率大幅度提高，单位地区生产总值建设用地占用面积大幅度下降，主要污染物排放总量大幅度削减，生产方式、生活方式和消费方式向绿色循环低碳转变，建成国家生态园林城市群。

　　——城乡功能品质显著提升。以人为核心的新型城镇化和城乡发展一体化步伐加快，公共资源配置更为均衡。城乡空间布局更趋优化，中心城市首位度继续提高，城市竞争力明显增强。与特大城市发展相适应的规划建设管理体系基本建立，现代综合交通设施更加完善，历史文化名城特色和江南水乡风貌充分彰显。

　　——文化繁荣程度显著提升。中国梦和社会主义核心价值观深入人心，向上向善、诚信互助的社会风尚广为弘扬，市民科学文化素质和社会文明程度同步提高。传统文化与现代文明交相辉映，文化事业与文化产业共同发展，文化影响力持续扩大，城市软实力明显增强。

　　——社会治理能力显著提升。“法治苏州”、“平安苏州”建设取得实效，法治政府、廉洁政府和服务型政府基本建成。社会公平正义得到保障，信用体系更加健全，社会治理现代化加快推进，人民安居乐业、社会和谐稳定的大好局面进一步稳固。

　　为了实现上述目标，建议新一届政府在工作中切实把握好以下五个方面：

　　一是更加注重创新驱动。回答好、落实好“创新四问”，加强以科技创新为核心的全面创新，积极发挥在全省创新格局中的引领性作用，努力冲在全国创新第一线。集聚高端要素，加强原始性创新，形成更多原创性成果。强化企业创新主体地位，促进企业家真正成为创新活动的主导者、创新资源的配置者和创新效益的获得者，打造苏州标志性品牌。突出开放性包容性，弘扬尊重知识、尊重人才、尊重创造的创新文化，构建人人皆可创新、创新惠及人人的良好创新生态系统。

　　二是更加注重普惠共享。切实解决人民群众最关心最直接最现实的利益问题，增强人民群众的获得感和幸福感，确保小康路上一个都不掉队。以就业为民生之本，以创业带动就业，促进城乡居民持续增收。坚持普惠性、保基本、均等化、可持续方向，增加公共产品供给，提高公共服务共建能力和共享水平。发展人民民主，保障人民平等参与、平等发展的权利。建设法治经济、法治政府、法治社会，使法治成为苏州核心竞争力的重要标志。

　　三是更加注重绿色宜居。把生态文明建设贯穿于经济社会发展各方面和全过程，坚持走生产发展、生活富裕、生态良好的发展道路，促进人与自然和谐。加大生态保护和环境治理力度，为人民群众提供更多优质生态产品。推动循环发展，集约利用资源，提高使用效率。建立系统完整的生态文明制度体系，构筑更为坚固的生态安全屏障。

　　四是更加注重统筹协调。正确处理经济与社会、城市与农村等重大关系，促进全面协调发展。针对经济社会发展中的薄弱环节，定向施策，精准发力，补齐短板。坚持工业反哺农业、城市支持农村，加快城乡发展一体化进程。优化城镇空间和产业布局，强化开发区增长极功能，推动区域协同发展。加强文化遗产保护性利用和传统工艺生产性挖掘，发挥历史文化资源的现代价值。在继承和弘扬优秀传统文化的基础上，推进文化创新，实现城市文脉永续传承。

　　五是更加注重双向开放。实施互利共赢的开放战略，更好利用国际国内两个市场、两种资源，形成以国际化企业为主体、国际化城市为基础、国际化人才为支撑的对外开放新格局。坚持引资和引技引智并重，提升开放载体平台建设水平，促进产业链向价值链高端攀升。积极投身“一带一路”、长江经济带和长三角世界级城市群建设，加强对外投资和产业协作，厚植开放型经济发展优势。

　　三、2017年的主要任务

　　2017年是党的十九大召开之年，也是新一届政府履职的第一年。根据中共苏州市委十二届二次全体（扩大）会议的部署，按照新一届政府工作的总体思路，在新的一年里，要坚持稳中求进工作总基调，以提高发展质量和效益为中心，以供给侧结构性改革为主线，全面做好稳定增长、创新驱动、民生改善、生态优化、改革开放、城乡建设等工作，促进经济平稳健康发展、社会和谐进步，以优异成绩迎接党的十九大胜利召开。做好今年工作，关键是牢牢把握稳中求进工作总基调。“稳”主要指稳住经济运行、稳住物价水平、稳住市场预期、稳住社会大局，坚决守住金融风险、社会民生、生态环境等底线。“进”主要指供给侧结构性改革、实体经济转型发展、产业科技创新、重点领域和关键环节体制改革、对外开放提质增效、民生实事项目建设取得新突破。

　　全市经济社会发展的主要预期目标是：地区生产总值增长7%～7.5%，一般公共预算收入增长8%左右，全社会固定资产投资与上年持平，实际使用外资60亿美元左右，全社会研究与试验发展经费支出占地区生产总值比重达到2.75%，企业研究与试验发展经费投入增长10%左右，万人有效发明专利拥有量增长12%，全员劳动生产率增长7.5%左右，工业增加值率提高0.5个百分点左右，居民人均可支配收入增长高于地区生产总值增幅，社会消费品零售总额增长10%左右，居民消费价格总水平涨幅控制在3%左右，新增就业15万人，城镇登记失业率控制在3%以内，单位地区生产总值能耗下降、主要污染物排放总量削减完成省下达任务。

　　1．强化创新驱动发展，增强产业竞争能力。落实支持科技创新的系列政策举措，实施创新发展十项重点工程，集聚创新资源，集中创新区域，打造创新高峰，提高科技进步贡献率。一是推进原始性创新。充分发挥企业创新主体作用，鼓励企业增加研发投入，建设高水平研发机构。加强产学研联合体建设，实施苏州—清华大学创新专项行动计划。聚焦微纳制造、第三代半导体、创新药物等领域，推进100个重大科技创新项目，突破关键技术，形成具有自主知识产权的拳头产品。二是集聚高端创新人才。落实人才政策，用好18亿元年度人才专项资金，引进培育1～2个拥有重大原创技术的创新创业团队，新增领军人才及团队500个、重点产业紧缺人才2000人、高技能人才20000人。实施企业家互联网思维提升计划，造就一支具有全球视野、开拓精神、创新能力的企业家队伍。办好国际精英创业周、技能英才周、“赢在苏州”海外创业大赛，建立海外人才离岸孵化平台。三是建设重大创新载体。推动高新技术产业开发区面向全球集聚和整合创新资源，建设集知识创造、技术创新和新兴产业培育为一体的创新核心区。争取吴江汾湖高新区、张家港高新区、太仓高新区升格为国家级高新区，常熟虞山高新园成为省级高新区。加快建设纳米真空互联实验站、干细胞实验平台、中国移动研发中心等重大创新平台，支持沙钢集团创建国家工程技术研究中心。落实企业创新能力提升和大企业（集团）培育计划，形成5家左右具有国际竞争力的创新型领军企业和世界影响力的品牌企业。四是优化创新生态系统。完善有利于创新的体制机制，充分释放创新红利。发展种子基金、天使基金、创业投资引导资金和产业投资资金，健全“科贷通”省市县三级联动机制，扩大科技信贷风险补偿资金规模，帮助1万家以上企业解决2000亿元融资需求。发挥高校的创新源头作用，支持在苏高校深度参与全市创新活动，吸引国内外著名高校在苏办学，加快建设昆山杜克大学，积极创办大学科技园和研究院。完善科技服务体系，发挥新兴产业标准化协作平台的引领和支撑作用，拓展国家技术转移苏南中心服务功能，畅通科技成果转移转化渠道。加强众创空间和虚拟创新社区等众创载体建设，探索政府与大企业共同创办创新创业载体。做好知识产权综合管理改革试点工作，建立支撑创新发展的知识产权运行机制。深化质量强市建设，扩大高质量产品和服务供给。

　　加快产业结构调整，推动产业转型升级，做优做强实体经济。一是大力发展高端产业。实施新兴产业跨越行动计划，按照每个县级市、区1～2个主攻方向的要求，布局和发展生物医药、新一代信息技术、纳米技术应用等战略性新兴产业，新兴产业产值占规模以上工业总产值比重提高1个百分点左右。建设国家级微纳制造创新中心、生物医药（医疗器械）产业园区和出口光电信息产业质量安全示范区。二是提档升级传统产业。实施智能制造“十百千万”工程，培育壮大以机器人为重点的智能装备产业。以智能制造为切入点，改造提升传统制造业。创建国家制造业与互联网融合发展试点城市。建立以亩均效益为核心的资源集约利用正向激励和反向倒逼机制，实行差别化的要素价格政策，促进资源节约高效利用。支持吴江纺织循环经济产业园建设。三是提升发展现代服务业。服务业增加值占地区生产总值比重提高1个百分点。发展金融、物流、中介服务等生产性服务业，推动制造业服务化，争创省级生产性服务业领军企业和集聚示范区。支持苏州高新区“金融小镇”建设，打响“太湖金谷”品牌。大力发展大数据、云计算、物联网、电子商务等新业态新模式，引进培育标杆企业，建设大数据特色产业园，做强新兴服务业。加快发展商贸流通、健康养老、社区便民服务等生活性服务业。建设文化创意产业基地，推动苏州工业园区争创国家级文化产业示范园区。以古城旅游为龙头，突出太湖旅游特色，整合旅游资源，丰富旅游业态，创建国家全域旅游示范区。启动建设环古城河与金鸡湖水上游等项目，推进文旅融合发展，以文化提升旅游内涵和品位，以旅游带动文化传播与繁荣。四是加快农业现代化步伐。推进农业供给侧结构性改革，抓好农产品标准化生产、品牌建设和质量安全监管，增加绿色优质农产品供给。强化物质装备和技术支撑，推动“互联网+现代农业”发展，促进主导产业提升、特色产业扩面、多元复合经营，新增现代农业园区面积4.67千公顷。创新农业经营机制，培育新型经营主体和社会化服务主体。完善农产品市场体系，建设苏州现代农产品物流园。做好第三次全国农业普查工作。五是优化实体经济发展环境。加大政策扶持力度，强化各类要素保障，推动金融资源与制造业企业有效对接，让更多金融活水流入实体经济。引导企业发扬“工匠精神”，支持企业走“专精特新”发展之路，不断提高生产效益、产品质量与核心竞争力，培育更多“百年老店”和细分行业的“单打冠军”。

　　2．注重供需两端发力，保持经济稳健运行。有力有效落实供给侧结构性改革任务，以创新供给带动需求扩张。一是积极稳妥去产能。完成省下达的钢铁去产能任务，削减300万重量箱平板玻璃产能，关停淘汰落后低效产能企业700家，兼并重组、帮扶解困企业70家，落实产业转移和“走出去”发展项目35个以上。二是分类施策去库存。完善商业办公用房去库存工作机制，对符合法律法规和规划要求的引导转为科技企业孵化器、众创空间、养老和旅游设施。加强调控监管，促进房地产市场平稳健康发展。三是严格管理去杠杆。加大股权融资力度，降低企业杠杆率，力争非金融企业直接融资占全社会融资比重达到30%。加强政府性债务管理，发挥资产管理公司作用，妥善处理不良资产，切实防范化解可能出现的风险隐患。四是优化服务降成本。落实减税降费政策，减少审批环节，降低企业交易成本特别是制度性交易成本以及融资、用能、物流等成本，全年减轻企业负担330亿元以上。五是精准有效补短板。围绕民生事业、公共服务等方面的薄弱环节，建立补短板项目库，完善补短板项目储备和推进机制，实施112个项目，完成投资220亿元。

　　积极扩大有效需求，着力释放新需求，确保经济平稳增长。一是增加有效投入。建设总投资超过1.1万亿元的210项重大项目，年内完成投资超过1360亿元。优化投资结构，重点投向新动能新产业培育和基础设施、生态环境、社会事业、农业水利等领域。鼓励企业增加技改投入，力争占工业投入比重提高1个百分点。二是扩大消费需求。促进服务消费、信息消费、网络消费、品牌消费、信用消费和休闲消费，开拓健康养老、教育培训、康复护理等服务，争创国家文化消费试点城市，加快推动消费升级。三是力促外贸增长。采取打造出口名牌、开拓多元市场、助推“互联网+外贸”、积极扩大进口等措施，努力实现对外贸易稳步发展。调整优化外贸结构，提档升级加工贸易，做优做强一般贸易。扩大服务贸易规模，争取服务贸易进出口增长15%。发展新型外贸业态，力争跨境电商、外贸综合服务、市场采购贸易等出口超过20亿美元。

　　3．不断加大惠民力度，增进人民群众福祉。持续增加民生投入，着力办好以“三优三清三提升”为主要内容的民生实事，让发展成果更多更公平惠及全市人民。一是促进城乡居民增收。将老百姓“口袋”鼓起来作为民生工作的重中之重，推动就业创业富民取得新成效，努力补齐民生短板中的收入短板。实施更加积极的就业政策，拓展就业空间，苏州籍高校毕业生就业率保持在95%以上，确保零就业、零转移家庭动态清零。健全工资正常增长机制，提高职工工资收入。千方百计推动创业，分类实施留学回国人员创新创业、大学生创业引领、农村创业富民、城镇失业人员创业促进四项计划，以创业带动增收。促进农村一二三产业融合发展，完善农村股权分配制度，增加农民经营性、财产性收入。二是健全社会保障体系。深入开展全民参保登记工作，巩固社保扩面成果。完善居民大病保险，发展商业养老保险和健康保险。推进居家和社区养老服务改革、医养融合发展、长期护理保险三项国家试点，提供优质养老服务，新增养老机构床位1000张，新建日间照料中心100家、助浴点20个。完善多层次救助体系，发展慈善事业，实施精准救助，确保困难群体应救尽救。实施残疾人就业增收、托底保障、服务普及、社会关爱工程，加快推进残疾人小康进程。三是改善群众“吃住行”条件。加强“菜篮子”基地和粮安工程建设，改造提升农贸市场。深入开展食品药品安全放心行动，建立源头可溯、环节可控、去向可查的监管体系。建设保障性安居工程，更好发挥住房公积金制度保障功能。推动轨道交通与常规公交融合发展，建设停车换乘设施，方便群众出行。加强市场价格调控和监管，保持物价水平基本稳定。四是创新和加强社会治理。深化“政社互动”、“三社联动”，发展社会组织和志愿者队伍，推进多元共治。健全社会信用体系，争创全国信用建设示范城市。广泛开展法治宣传教育和法治惠民活动。进一步做好民族宗教工作。构建信访工作责任体系，及时回应群众诉求。落实安全生产企业主体责任和政府监管责任、部门工作职责，狠抓重点领域专项治理，加大重大隐患整改挂牌督办力度，坚决遏制重特大事故。完善社会综合治理联动机制，强化网格化管理，夯实基层基础，严密防范、依法打击违法犯罪活动，力争实现全国社会治安综合治理优秀市“七连冠”、长安杯“四连捧”。支持国防和军队改革，落实拥军优属政策，推动军民融合发展，争创全国双拥模范城“七连冠”。

　　4．切实抓好生态保护，营造优美宜居环境。牢固树立“绿水青山就是金山银山”的理念，实施生态文明建设630行动计划，开展“两减六治三提升”和沿江化工整治专项行动，突出抓好十项重大工程，年内完成投资100亿元。一是实施清水行动计划。开展新一轮东太湖整治、长江流域治理和阳澄湖新三年生态优化行动，轮浚城乡河道，整治黑臭水体，完成1000个村庄生活污水治理任务，启动苏州中心城区清水工程，全面推行河长制。整治规范畜禽养殖。二是实施蓝天行动计划。落实大气污染防治计划，治理挥发性有机物，完成石油炼制、石油化工、合成树脂行业50家企业的提标改造和化工园区50家企业的泄露检测修复，完成剩余6台10万千瓦以上燃煤机组改造和30家工业企业码头堆场扬尘整治任务，扩大高污染燃料禁燃区范围，降低PM2.5浓度，增加空气质量优良天数。实施重点耗能企业节能改造，提高清洁能源使用比重，建设国际能源变革发展典范城市。三是实施净土行动计划。出台国家“土十条”苏州实施方案，加强土壤环境质量监测和污染防治，切实保障土壤安全。重点治理修复原苏州溶剂厂、化工厂厂址等污染地块。四是实施化工行业整治行动计划。加快淘汰落后和搬迁入区进程，关停并转化工集中区和工业集中区以外的化工生产企业，提高区内企业安全生产和环境保护水平。五是实施生态修复和保护行动计划。强化生态功能区管控，加大湿地保护力度，建设山水林田湖生态保护带，完成森林抚育2.67千公顷。推进城市绿化提升工程，优化绿化景观格局，市区新增绿地400万平方米。六是实施环保能力提升行动计划。加快建设七子山生态修复工程。扩大生活垃圾分类收集处置覆盖面，促进再生资源回收利用。强化环保监测监察，增强监管执法能力。

　　5．持续深化改革开放，增添发展动力活力。以全局观念和系统思维谋划改革举措，着力破除制约经济社会发展的体制机制障碍。一是推进“放管服”改革。建立以权责清单为核心的阳光高效审批制度，实施精准协同放权。深入开展相对集中行政许可权改革试点工作，选择部分县级市、开发区和经济发达镇推广苏州工业园区集中审批试点经验，筹建市级行政审批局。加强事中事后监管，完善以信用为基础的综合有效监管执法体系，实现“双随机、一公开”监管全覆盖。建成“互联网+政务服务”体系，开展“减证便民”专项行动，最大程度方便群众和企业。二是深化商事制度改革。落实企业“多证合一、一照一码”和个体工商户“两证合一”等改革措施，推行全程电子化登记、电子营业执照应用、登记信用承诺等创新举措。三是深入实施投融资体制改革。推广政府和社会资本合作、特许经营等投融资模式，鼓励民间资本参与基础设施、生态环保和社会事业等领域投资。探索投贷联动、债贷结合等改革。促进金融机构开展普惠金融业务，拓宽保险资金参与基础设施建设和支农支小渠道。四是发展壮大市场主体。深化国企国资改革，加快形成有效制衡的法人治理结构、灵活高效的市场化经营机制。大力推动民营经济发展，放宽市场准入，消除隐形壁垒，形成更加公平有序、透明高效的营商环境，使民营经济成为全市经济的主要支柱。积极发展混合所有制经济，鼓励民营企业参与国有企业改革，鼓励国有企业投资项目引入非国有资本。加强对各种所有制组织和自然人财产权的保护，稳定社会预期，增强发展信心。弘扬企业家精神，激发全社会创新创业的热情和活力。

　　实施开放型经济创新提升“七大工程”，提高对外对内开放的质量和效益。一是加快推动先行先试。力争苏州工业园区叠加自由贸易试验区政策取得突破。做好跨境电子商务、服务贸易创新发展、市场采购贸易和赋予综合保税区内企业增值税一般纳税人资格等试点工作，努力实现政策优势互补和全市共享。二是精心打造开放平台。深化苏州工业园区开放创新综合试验和昆山深化两岸产业合作试验区改革，全力创建中德产业合作创新试验区，加快形成对外开放三大平台。推动苏相合作经济开发区发展，支持相城区设立综合保税区。三是更高水平“引进来”。进一步降低外资准入门槛，全面实施外商投资准入前国民待遇加负面清单管理制度。鼓励外资以特许经营方式参与基础设施建设，支持外资企业在国内上市、发债。强化产业链、价值链中高端招商，重点引进外资研发机构和地区功能性总部。四是更大步伐“走出去”。培育壮大跨国经营主体，支持有条件的企业面向全球布局创新网络，并购具有自主知识产权的知名企业和品牌。加强同“一带一路”沿线国家和地区的产能合作与经贸往来，推进埃塞俄比亚东方工业园建设，推动“苏满欧”等国际铁路货运班列发展，争取铁路二类口岸早日获批。加强外事、对台和侨务工作，促进经贸合作与人文交流。建设布达佩斯中国文化中心。五是持续扩大对内开放。深度融入长江经济带建设，加强与中上游地区、长三角城市的合作。建好太仓港长江水运集散中心，启动太仓港集装箱码头四期工程。深化南北挂钩合作，做好对口支援帮扶工作。

　　6．统筹推进建设管理，提升城乡发展水平。围绕融古铸今、充满活力、生态宜居、特色鲜明的城市发展要求，建设重大工程，完善功能，提升品质。一是科学编制各类规划。做好苏州城市总体规划（2030）、综合交通体系规划编制前期工作。创新规划理念和方法，以主体功能区规划为基础统筹各类空间性规划，划定各县级市、区和镇开发边界，推进“多规融合”。精心开展城市设计，彰显苏州城市特色。二是加强历史文化名城保护。做好整体保护、特色塑造、业态转型、品质提升、民生改善等文章，当好古城的守护者和传承人。推进历史文化名城保护和提升六大工程，实施古城保护示范、违法建设专项整治、老旧住宅小区和背街小巷整治、城中村环境综合整治、交通综合整治、河道截污清水等项目。深入推进虎丘地区综合改造、桃花坞片区综合整治保护利用、“两河一江”综合整治和古建老宅保护修缮等工程，沟通城市肌理，延续历史文脉。系统保护古镇古村落和各类文化遗产，继续做好江南水乡古镇和海上丝绸之路申遗工作，建立苏州戏曲传承中心，扩大手工艺与民间艺术之都、世界遗产城市和吴文化中心的品牌影响力。三是构建综合交通体系。加快建设沪通铁路苏州段，力争南沿江城际铁路早日开工，做好通苏嘉城际铁路、沪苏湖城际铁路、太仓港疏港铁路前期工作。建成沪宁高速公路昆山高新区互通工程，改扩建524国道常熟段和相城段、锡通过江通道公路南连接线。推进杨林塘等航道整治。建成城市轨道交通4号线及支线，新增运营里程52.8公里，建设3号线、5号线，力争开工6号线和市域S1号线；苏州高新区有轨电车2号线及1号线延伸线投入试运营。四是推进城市有机更新。加强城市修补和生态修复，着力补齐功能短板。加快建设苏州太湖新城、高铁新城。基本建成澄阳路、城北路、太湖新城地下综合管廊。实施京杭大运河苏州段岸堤加固和景观提升工程。建设海绵城市，提标改造城市排水防涝设施。新增人防设施120万平方米，增建应急避难场所。推动1000千伏特高压淮上线输变电工程苏通管廊建设。下大力气治理建成区违法建筑。制定实施“城市云”建设规划，提升“智慧城市”服务保障能力。建立智慧城管大数据平台和智慧环卫监管平台，整合运用市容环境、交通运行等数据资源，提高管理精细化、可视化水平。

　　统筹推进新农村建设，持续兴办农村实事，让乡村生活更加美好。一是深化城乡发展一体化综合改革。推动农村土地三权分置改革，开展农民土地承包经营权抵押试点，探索土地承包经营权有偿退出的路径和办法，组建农村产权交易分中心。加强农村金融服务，创新试点重点农产品价格指数保险。深入开展“三优三保”行动，新增试点镇10个。二是发展壮大农村集体经济。加强农村集体资产监管，健全集体资产管理营运体系，引导新型集体经济组织联合发展、异地发展，农村集体总资产增长6%。三是切实加强美丽镇村建设。推进美丽城镇建设，实施特色小镇培育计划，完成14个被撤并镇综合整治提升任务。启动新一轮美丽村庄建设行动，建设10个康居特色村和350个三星级康居乡村。完成市域内铁路、高速公路和内河主航道沿线绿化及环境专项整治。

　　7．加快发展社会事业，优化基本公共服务。推动公共资源向基层延伸、向农村覆盖、向弱势群体倾斜，提升公共服务水平。一是促进教育优质均衡多样发展。新建和改扩建中小学、幼儿园77所，建设未成年人社会实践体验活动站综合服务平台。做好流动人口随迁子女积分入学工作，提档升级外来工子女学校。坚持面向市场、服务发展、促进就业的办学方向，发展现代职业教育。实施名城名校融合发展战略，提高在苏高校省市共建水平。二是推进“健康苏州”建设。确立健康优先理念，实施健康市民、健康城市、健康卫士系列行动计划。深化国家公立医院改革和省级医药卫生体制改革试点，完善分级诊疗体系，提高医疗卫生服务水平。建设市独墅湖医院、中医院二期工程，建成市公共医疗中心和第九人民医院。建设体育训练基地、体育主题公园和健身步道等各类设施，构建“10分钟体育休闲生活圈”。三是丰富群众精神文化生活。践行社会主义核心价值观，继承张家港精神、昆山之路、园区经验“三大法宝”，弘扬新时期苏州精神。深入开展文明创建活动，力争苏州市实现全国文明城市“四连冠”，张家港市实现“五连冠”，常熟市、太仓市进入全国文明城市行列。做好修志编鉴工作。完善公共文化服务体系，建设苏州第二图书馆、第二工人文化宫和工业展览馆、名城水文化馆，完成400个村、社区综合性文化服务中心标准化建设任务。

　　8．全面加强自身建设，打造高效清廉政府。牢固树立政治意识、大局意识、核心意识、看齐意识，严守政治纪律和政治规矩，坚决维护党中央权威，确保政令畅通、一贯到底。坚持为民务实清廉，忠实履行政府职责，建设人民满意政府。一是牢记为民宗旨。坚持以人民为中心的发展思想，把执政为民贯穿到政府工作的各领域全过程。深化领导干部下基层“三访三促”活动，切实为基层、企业、群众排忧解难。增强服务意识，推进行政审批、公共资源交易、便民服务一体化建设，主动为群众和企业提供优质服务。二是严格依法行政。落实政府权力和责任清单，切实做到法无授权不可为、法定职责必须为。认真执行重大行政决策程序规定，确保决策制度科学、程序正当、过程公开、责任明确。推进城市综合执法，完成市、县级市（区）两级城市管理领域执法机构综合设置。健全行政复议、行政调解制度，做好行政复议案件办理、行政诉讼案件出庭应诉等工作。深化政务公开，加强信息发布和政策解读，及时回应社会关切。三是切实改进作风。不折不扣贯彻执行作风建设各项规定，持续转作风提效能。完善考核评价体系，建立正向激励机制，营造想干事、能干事、干成事的良好氛围，提振政府工作人员、企业家和各类人才干事创业的精气神。加强学习，深入调研，增强工作预见性，超前谋划重点工作、重大项目。本着“一市（区）一题”的思路，帮助各县级市（区）每年解决一个事关长远发展的重大问题。坚决防止“四风”反弹回潮，严肃查处发生在群众身边的不正之风。四是坚持廉洁从政。认真落实党风廉政建设责任制，严格执行党章党规，强化纪律刚性约束。坚持无禁区、全覆盖、零容忍，有腐必反、有贪必肃，始终保持惩治腐败高压态势。主动接受人大法律监督、工作监督和政协民主监督，接受司法机关监督，接受社会公众和新闻舆论监督。推进公共资金、国有资产、国有资源和领导干部履行经济责任情况审计监督全覆盖，探索领导干部自然资源资产离任审计，强化政策执行和民生环保审计。认真做好人大议案、代表建议和政协提案办理工作，提高办理水平和质量。

　　各位代表，时代赋予重任，人民寄予厚望。让我们更加紧密地团结在以习近平同志为核心的党中央周围，高举中国特色社会主义伟大旗帜，在中共苏州市委的坚强领导下，团结一心，开拓进取，为高水平全面建成小康社会、争当建设“强富美高”新江苏先行军排头兵、谱写好伟大中国梦的苏州篇章而努力奋斗！
